# An observational descriptive study of the epidemiology and treatment of neuropathic pain in a UK general population

**DOI:** 10.1186/1471-2296-14-28

**Published:** 2013-02-26

**Authors:** Gillian C Hall, Steve V Morant, Dawn Carroll, Zahava L Gabriel, Henry J McQuay

**Affiliations:** 1 Grimsdyke House, Ravenscroft Park, London, EN5 4ND, UK; 2 Independent Statistician, Hadenham, Bucks, UK; 3 Outcomes Research and Evidence Based Medicine, Pfizer Ltd, Tadworth, UK; 4 Emeritus Fellow Balliol College, University of Oxford, Oxford, UK

**Keywords:** Neuropathic pain, Incidence, Post-herpetic neuralgia, Painful diabetic neuropathy, Phantom limb pain, Treatment, Antidepressant, Antiepileptic, Primary care

## Abstract

**Background:**

This study updated our knowledge of UK primary care neuropathic pain incidence rates and prescribing practices.

**Methods:**

Patients with a first diagnosis of post-herpetic neuralgia (PHN), painful diabetic neuropathy (PDN) or phantom limb pain (PLP) were identified from the General Practice Research Database (2006 – 2010) and incidence rates were calculated. Prescription records were searched for pain treatments from diagnosis of these conditions and the duration and daily dose estimated for first-line and subsequent treatment regimens. Recording of neuropathic back and post-operative pain was investigated.

**Results:**

The study included 5,920 patients with PHN, 5,340 with PDN, and 185 with PLP. The incidence per 10,000 person-years was 3.4 (95% CI 3.4, 3.5) for PHN; and 0.11 (95% CI 0.09, 0.12) for PLP. Validation of the PDN case definition suggested that was not sensitive. Incident PHN increased over the study period. The most common first-line treatments were amitriptyline or gabapentin in the PDN and PLP cohorts, and amitriptyline or co-codamol (codeine-paracetamol) in PHN. Paracetamol, co-dydramol (paracetamol-dihydrocodeine) and capsaicin were also often prescribed in one or more condition. Most first-line treatments comprised only one therapeutic class. Use of antiepileptics licensed for neuropathic pain treatment had increased since 2002–2005. Amitriptyline was the only antidepressant prescribed commonly as a first-line treatment.

**Conclusion:**

The UK incidence of diagnosed PHN has increased with the incidence of back-pain and post-operative pain unclear. While use of licenced antiepileptics increased, prescribing of therapy with little evidence of efficacy in neuropathic pain is still common and consequently treatment was often not in-line with current guidance.

## Background

A fifth of adults in Europe report moderate or severe chronic pain with 2% managed by a specialist [1]. Neuropathic pain is defined as pain arising as a direct consequence of a lesion or a disease affecting the somatosensory system [2]. The prevalence of pain of predominantly neuropathic origin has been reported as 7–8% in French and UK surveys [3,4], and is more intense in comparison with chronic pain without neuropathic characteristics [4]. The incidence of any neuropathic pain has been reported as 0.8% per annum in a Dutch primary care database [5], while two previous UK studies based on electronic primary care records showed that the incidence of both post-herpetic neuralgia and phantom limb pain had been decreasing, while incident painful diabetic neuropathy remained stable (1992 to 2005) [6,7]. The rate of diagnosed painful diabetic neuropathy may have subsequently increased as UK primary care physicians are now encouraged to ask diabetic patients about neuropathic symptoms [8].

A 2006 European task force on pharmacological treatment of neuropathic pain reported that treatment remained unsatisfactory despite a substantial increase in the number of clinical trials [9]. These trials provide evidence for the efficacy of tricyclic antidepressants, the antiepileptic drugs gabapentin, pregabalin and opioids (in post-herpetic neuralgia and painful polyneuropathies), with some evidence on the efficacy of topical lidocaine (in post-herpetic neuralgia) and the antidepressants, venlafaxine and duloxetine (in painful polyneuropathies) [10]. In UK primary care between 1999–2002 and 2002–2005, treatment of neuropathic pain conditions shifted from predominantly non-opioid analgesics towards tricyclic antidepressants and antiepileptics [6,7]. That change may be due to a combination of substantial increase in evidence, education and the introduction and marketing of drugs licensed for use in this indication.

Neuropathic back pain and post-operative pain other than phantom limb pain were not studied in the previous UK primary care database analyses, as the identification of these less well defined neuropathic pain conditions was expected to be more difficult from a primary care database. However, these conditions affect large populations. The adult annual rate for consulting general practice for back pain is 6.4% [11], of which 16% may have possible neuropathic pain [12]. Post-operative pain is also common, although incidence rates vary between studies. For example, women who undergo breast surgery are reported to experience chronic chest wall, breast, or scar pain (range, 11–57%), phantom breast pain (13–24%), and arm and shoulder pain (12–51%) while the mean incidence of chronic pain after inguinal hernia surgery was estimated as 11.5% [13].

The objectives of this study were to update the incidence rates and prescribing practices for post-herpetic neuralgia, phantom limb pain and painful diabetic neuropathy in UK primary care and to report any time trends, and to examine the recording and treatment of neuropathic back pain and post-operative pain.

## Methods

### Data source

The source population and prescribing data were retrieved from the General Practice Research Database (GPRD). The GPRD is an observational database containing information collected in computerised primary care practices throughout the UK. Details of demographics, primary care diagnoses and prescription treatment are routinely recorded against date in individual patient records. Details of referrals, secondary care diagnoses and deaths are also captured because of the structure of the UK National Health Service. Major events from before computerisation are added retrospectively. Data on preventive medicine can also be recorded. Medical events are automatically coded at entry using the Read coding system [14]. Each patient on GPRD is given an ‘up to standard’ date when their record is considered to be of sufficient quality for research. A total of 531 practices were included in the study. The protocol was approved by the GPRD scientific and ethics committee.

### Cohort definitions

The study population comprised all patients who were permanently registered at a GPRD practice at any time in the study period, from 1st January 2005 to 31^st^ December 2010. Five neuropathic pain cohorts (post-herpetic neuralgia, painful diabetic neuropathy, phantom limb pain, neuropathic back pain and neuropathic post-operative pain) were identified from this study population by searching individual patient records for either a single specific Read code, or a combination of Read and therapy codes as specified in a case definition. Post-herpetic neuralgia was defined as a specific code for post-herpetic neuralgia, or a code for acute zoster plus either a code for neuropathy, or neuropathic pain, between three and six months after the first acute zoster entry. Phantom limb pain (PLP) was defined as a specific code, or a code for amputation plus either a code for neuropathy or neuropathic pain between three and twenty-four months after the first amputation code. The painful diabetic neuropathy cohort included patients with a specific code for painful diabetic neuropathy; those with a code for diabetes and a general code for neuropathic pain and a third group with a code for diabetic neuropathy (or diabetes and neuralgia) with a prescription for a neuropathic pain treatment which was initiated within 28 days of the date of the neuropathy/neuralgia code.

There is no specific code for neuropathic back pain in the Read dictionary. The condition was defined as a code for back pain plus either a code for neuropathy or neuropathic pain within 28 days, or a code for radiculopathy or back pain with a specific neuropathic pain treatment (rather than general pain) initiated within 28 days of the back pain date. To identify patients with neuropathic post-operative pain preliminary database searches looked for patients with codes for surgery followed by a record of post-operative pain, or surgery plus either a code for neuropathic pain, or neuropathy or persistent pain and a prescription for a specific neuropathic pain treatment (rather than general pain) initiated within 28 days of the pain record date. Few patients were found to meet these criteria, so to investigate the recording of this condition, patients with a code for breast or hernia surgery were identified as the prevalence of pain has been reported in this group [13]. For this post-operative sub-group, records in the three to six months after surgery were searched to identify patients with a code for neuropathic pain, a code for post-operative pain plus a code for neuropathy within 28 days, or a code for neuropathy or post-operative pain plus a prescription for a specific neuropathic pain treatment (rather than general pain) initiated within 28 days of the neuropathy/pain code.

The cohort entry date for each patient was the date of the first Read code for the specific condition (phantom limb pain, painful diabetic neuropathy or post-herpetic neuralgia), neuropathic pain, neuropathy, neuralgia, or post-operative pain in the case definition. Prevalent cases with a cohort entry date before 1^st^ January 2006 were excluded. Patients could be included in more than one cohort, but not in both phantom limb pain and post-operative pain. A period of observation was set for each subject with a start date of 1^st^ January 2005 or, if later, the date that the patient record was suitable for research plus twelve months. The twelve months was added to allow time for the recording of prevalent conditions before the study started. The end date was the first of the following: the end of the study period, death, transfer-out of the practice, or the final data collection.

### Validation of diagnosis

Questionnaires were sent to the general practitioners (GPs) of a random sample of patients to confirm the diagnosis. The sample comprised 108 patients with post-herpetic neuralgia and 54 patients within the study definition of painful diabetic neuropathy. An additional group of 52 questionnaires tested for false negatives among those patients whose records did not meet the case definition but who did have a record of diabetes and treated neuropathy; for example when a diabetic patient had peripheral neuropathy but was already on prescription treatment for an analgesic at the time of the first mention of peripheral neuropathy.

### Prescription neuropathic pain therapy

A neuropathic pain treatment was defined as a prescription for an analgesic (excluding aspirin 100 mg or less), an anaesthetic (oral, dermal or intravenous), an antiepileptic (with no history of epilepsy), or an antidepressant. Therapeutic class was based on the British National Formulary (BNF) [15]; antiepileptics (BNF 4.8.1), antidepressants (BNF 4.3 in total and divided into 4.3.1 and other), analgesics, (BNF 4.7.1 non-opioid analgesics and 4.7.2 opioid analgesics), rubefacients - capsaicin (BNF 10.3.2), local anaesthesia including lidocaine hydrochloride (BNF 15.2) and other. Specific neuropathic pain treatments were those antiepileptics, antidepressants and opioids commonly used for neuropathic pain (Additional file 1).

### Treatment patterns

The details of prescriptions for neuropathic pain treatments were identified for the cohorts. For post-herpetic neuralgia, painful diabetic neuropathy and phantom limb pain the first-line treatment was defined as a neuropathic pain treatment started within 28 days of the cohort entry date. The case definitions for neuropathic back pain and post-operative pain included only those treatments specifically recommended or indicated in neuropathic pain which might be second-line treatments. Earlier prescribing was included in the treatment analysis in order to include first-line treatment with less specific agents. The first-line treatment for neuropathic back pain was therefore defined as the first neuropathic pain treatment in a treatment episode that spanned the cohort entry date, but did not start before the back pain or radiculopathy code, or that started in the 28 days after the back pain or radiculopathy code.

Daily dose was estimated from the details on the prescription and the duration of each prescription was estimated from the daily dose and number of items prescribed. When a prescription described a dose titration, then the final dose was included. When the duration could not be calculated from details on a prescription, it was assumed to last 28 days. If more than one therapy was prescribed on the same day then the first-line treatment was considered to be the combination of these therapies. If more than one therapy was prescribed, but on different days, the first-line treatment regimen included only items on the first prescription.

Duration of the first-line treatment was the sum of the individual prescription durations until discontinuation, or prescription, of one or more treatments. A second-line treatment regimen started when any change was made to the original regimen without a break in all treatment as a break in treatment was assumed to indicate a new episode of pain. The change could be prescription of a new treatment or discontinuation of part of the first-line treatment. A therapy was considered to have been discontinued when no additional prescription was issued within 62 days of the end of the last prescription. Third-line treatment was defined in the same way as any addition or discontinuation without a break in treatment.

### Analysis

The incidence per person-years observation for 2006 to 2010 was estimated for post-herpetic neuralgia, phantom limb pain and painful diabetic neuropathy. A rate (rather than incidence) of identified new cases of neuropathic back pain was estimated as the case definition for this condition was not specific. Those with a first record of their neuropathic pain dated during their period of observation were counted as incident cases. The denominator (person-years observation) was the sum of the difference between the start date plus one year and end date for each patient. The age and sex distribution at cohort entry was estimated assuming a date of birth of 1^st^ July of the recorded year of birth. Incidences were standardised to the 2010 UK age-sex distribution [16].

Descriptive analyses reported the number of patients with an first-line treatment identified, the treatments prescribed (including number and type of therapy and mean duration) and the percentage of use as first, second and third line therapy for treatments prescribed to more than 100 patients at any stage. The analysis was completed by condition for patients with at least 18 months prescribing data after the cohort entry date to provide sufficient prescribing history. The number of patients with each daily dosing regimen was counted by year for all first prescriptions for gabapentin or pregabalin.

## Results

### Incidence

**Figure 1:**
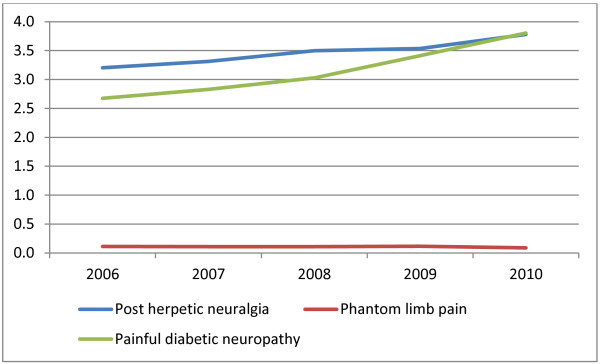
The annual incidence of neuropathic pain conditions (per 10,000 person-years) between 2006 and 2010.

The study cohorts included 5,920 patients with post-herpetic neuralgia, 5,340 with painful diabetic neuropathy, 185 with phantom limb pain and 90,941 with neuropathic back pain. This provided annual incidence rates per 10,000 population of 3.4 (95% Confidence intervals (CI) 3.4, 3.5), for post-herpetic neuralgia, 3.1 (95%CI 3.0, 3.2) for painful diabetic neuropathy and 0.1 (95%CI 0.09, 0.12), for phantom limb pain. When standardised to 2010 UK age and sex distribution, the annual incidence per 10,000 population were 3.0 (95%CI 3.0, 3.1) for post-herpetic neuralgia, 2.8 (95%CI 2.7, 2.8) for painful diabetic neuropathy and 0.11 (95%CI 0.09, 0.12) for phantom limb pain. The incidence of these three conditions increased with age (Table 1). Phantom limb pain was more common in men than women, while post-herpetic neuralgia was more common in women. The incidence of post-herpetic neuralgia and painful diabetic neuropathy increased over the study period (Figure 1). The mean annual number of patients with incident events within our case definition of neuropathic back pain was 18,188, increasing over the study period from 49 to 62 per 10,000 population, per annum (mean 53). Despite widening the original case definition, only 49 patients were identified as having post-operative neuropathic pain after either breast or hernia surgery. The treatment analysis for this group is therefore not reported.

**Table 1:** Annual incidence of neuropathic pain conditions per 10,000 by age and sex

		**Female**	**Male**
		**Incidence (95%CI)**	**Incidence (95%CI)**
**Post-herpetic neuralgia**	All ages	4.3 (4.2-4.4)	2.6 (2.5-2.7)
	0 - 14	0.1 (0.1-0.2)	0.1 (0.0-0.1)
	15 - 29	0.6 (0.4-0.7)	0.2 (0.2-0.3)
	30 - 44	1.0 (0.8-1.1)	0.5 (0.4-0.6)
	45 - 59	3.0 (2.8-3.3)	1.9 (1.7-2.1)
	60 - 74	9.2 (8.7-9.7)	6.6 (6.2-7.1)
	≥75	16.6 (15.8-17.5)	13.6 (12.7-14.6)
**Phantom limb pain**	All ages	0.1 (0.0-0.1)	0.2 (0.1-0.2)
	0 - 14	0	0
	15 - 29	<0.1 (0.0-0.1)	<0.1 (0.0-0.1)
	30 - 44	<0.1 (0.0-0.1)	0.1 (0.1-0.2)
	45 - 59	0.1 (0.0-0.1)	0.1 (0.1-0.2)
	60 - 74	0.1 (0.1-0.2)	0.4 (0.3-0.5)
	≥75	0.1 (0.1-0.2)	0.7 (0.5-0.9)
**Painful diabetic neuropathy cohort**	All ages	2.9 (2.8-3.0)	3.3 (3.2-3.4)
	0 - 14	0	<0.1 (0.0-0.1)
	15 - 29	0.2 (0.1-0.3)	0.1 (0.0-0.1)
	30 - 44	0.8 (0.7-1.0)	0.9 (0.8-1.0)
	45 - 59	3.0 (2.8-3.3)	3.7 (3.4-4.0)
	60 - 74	7.0 (6.6-7.5)	9.2 (8.7-9.7)
	≥75	8.3 (7.7-8.9)	10.8 (10.0-11.7)

### Validation of incidence

The GP questionnaire showed a positive predictive value of 91% for post-herpetic neuralgia (n = 86, 80% response) and 56% for painful diabetic neuropathy (n = 48, 89% response). In those patients who did not meet the full case definition for painful diabetic neuropathy 7% were identified as false negatives (n = 45, 87% response).

### Treatment

Across the study conditions, between 80 and 87% of patients had an available follow-up period of 18 months and so were included in the prescription treatment analysis (Table 2). The number with a first-line prescribed therapy identified ranged from 59% with phantom limb pain, to 100% with neuropathic back pain. A first-line therapy could have been missed in patients with antiepileptics and epilepsy, or if treatment was started more than 28 days before the date of the first record of the condition. The mean duration of the first therapy ranged from 48 to 121 months between conditions, but there was wide variation (Table 2). The majority of patients were prescribed one item initially. The most common first-line treatment was amitriptyline in post-herpetic neuralgia and the painful diabetic neuropathy cohort, gabapentin in phantom limb pain and tramadol in neuropathic back pain.

**Table 2:** First-line treatment by pain condition (2006–2010)

	**Post-herpetic neuralgia**	**Painful diabetic neuropathy**	**Phantom limb pain**	**Neuropathic back pain**
**Total**^**a **^**(% of cohort)**	4,725 (79.8)	4,317 (80.1)	153 (82.7)	78,693 (86.5)
**Number with a treatment (% total in analysis)**	3,831 (81.1)	3,513 (81.4)	90 (58.8)	78,417 (99.6)
**Duration of treatment (days), mean (±SD) **^**b**^	48 (115)	121 (229)	63 (86)	89 (229)
**Items prescribed on initial prescription as% of those treated**				
**1 item**	65.0	82.4	73.3	63.0
**2 items**	28.7	15.1	20.0	28.6
**3 or more items**	6.4	2.5	6.7	8.4
**Five most common medications included in first-line treatment regimens, n (% treated)**				
**1**	amitriptyline	amitriptyline	gabapentin	tramadol
	1509 (39.4)	1362 (38.8)	22 (24.4)	26,778 (34.1)
**2**	co-codamol	gabapentin	amitriptyline	amitriptyline
	754 (19.7)	539 (15.3)	19 (21.1)	17,093 (21.8)
**3**	capsaicin	co-codamol	paracetamol	co-codamol
	328 (8.6)	332 (9.5)	12 (13.3)	16,198 (20.7)
**4**	gabapentin	pregabalin	pregabalin	paracetamol
	327 (8.5)	315 (9.0)	12 (13.3)	6750 (8.6)
**5**	co-dydramol	paracetamol	tramadol	co-dydramol
	293 (7.6)	264 (7.5)	7 (7.8)	5320 (6.8)

^a^with incident disease plus 18 months record. ^b^ignoring titration of dose. SD, standard deviations.

An antidepressant or an antiepileptic was prescribed as part of a first-line treatment for 57.0% of post-herpetic neuralgia patients, 70.5% of the painful diabetic neuropathy cohort and 61.1% of the phantom limb pain cohort. Analgesics alone (opioid +/− non-opioid) were prescribed first-line for 36.3% of patients with post-herpetic neuralgia, 25.4% of patients in the painful diabetic neuropathy cohort and 37.7% of patients with phantom limb pain (Table 3). An opioid, or a combination of opioid and non-opioid analgesic, was prescribed most frequently as a first-line treatment for neuropathic back pain, 64.0% of cases (Table 3). Antiepileptics were the most common therapeutic category in first-line treatments prescribed for phantom limb pain. Most first-line treatments included only one therapeutic class.

**Table 3:** Initial treatments by condition and combination of therapeutic class

	**Post-herpetic neuralgia**	**Painful diabetic neuropathy**	**Phantom limb pain**	**Neuropathic back pain**
	***n (%)***	***n (%)***	***n (%)***	***n (%)***
**Total treated**	3,831 (100)	3,513 (100)	90 (100)	78,417 (100)
**Anaesthetics alone**	4 (<0.1)	1 (<0.1)	0	3 (<0.1)
**Antiepileptics alone**	526 (13.7)	892 (25.4)	27 (30.0)	3,030 (3.9)
**Non-opioid analgesics alone**	223 (5.8)	225 (6.4)	10 (11.1)	3,622 (4.6)
**Opioid analgesics alone**	296 (7.7)	254 (7.2)	17 (19.0)	25,903 (33.0)
**Other antidepressants alone**	23 (0.6)	79 (2.2)	0	3,007 (3.8)
**Rubefacients alone**	220 (5.7)	142 (4.0)	1 (1.1)	666 (0.8)
**Tricyclic antidepressants alone**	1,223 (31.9)	1,328 (37.8)	16 (17.8)	13,901 (17.7)
**Anaesthetics + tricyclic antidepressants**	1 (<0.1)	0	0	0
**Antiepileptics + non-opioid analgesics**	9 (0.2)	9 (0.3)	1 (1.1)	104 (0.1)
**Antiepileptics + opioid analgesics**	19 (0.5)	19 (0.5)	3 (3.3)	224 (0.3)
**Antiepileptics + other antidepressants**	4 (0.1)	5 (0.1)	1 (1.1)	84 (0.1)
**Antiepileptics + rubefacients**	13 (0.3)	9 (0.3)	0	9 (<0.1)
**Antiepileptics + tricyclic antidepressants**	10 (0.3)	7 (0.2)	2 (2.2)	100 (0.1)
**Non-opioid analgesics + other antidepressants**	0	0	0	133 (0.2)
**Non-opioid analgesics + rubefacients**	5 (0.1)	1 (<0.1)	0	44 (<0.1)
**Non-opioid analgesics + tricyclic antidepressants**	34 (0.9)	16 (0.5)	0	567 (0.7)
**Opioid analgesics + non-opioid analgesics**	873 (22.8)	412 (11.7)	7 (7.8)	20,651 (26.3)
**Opioid analgesics + other antidepressants**	0	3 (0.1)	2 (2.2)	358 (0.5)
**Opioid analgesics + rubefacients**	7 (0.2)	2 (0.1)	0	26 (<0.1)
**Opioid analgesics + tricyclic antidepressants**	54 (1.4)	21 (0.6)	0	1,147 (1.5)
**Other antidepressants + tricyclic antidepressants**	1 (<0.1)	4 (0.1)	0	120 (0.2)
**Rubefacients + tricyclic antidepressants**	55 (1.4)	11 (0.3)	0	20 (<0.1)
**1 therapeutic class**	2,515 (65.6)	2.921 (83.1)	71 (78.9)	50,132 (63.9)
**2 therapeutic classes**	1,085 (28.3)	519 (14.8)	16 (17.8)	23,590 (30.1)
**3 or more therapeutic classes**	231 (6.0)	73 (2.1)	3 (3.3)	4,695 (6.0)
**Antidepressant or antiepileptic**	2184 (57.0)	2,475 (70.5)	55 (61.1)	27,319 (34.8)
**Analgesic alone (opioid or non-opioid)**	1,392 (36.3)	891 (25.4)	34 (37.8)	50,176 (64.0)

The total number of patients who received a prescription for either gabapentin or pregabalin increased over the study period (Table 4). Gabapentin was prescribed most frequently with a dosage regimen of three times per day, whereas pregabalin was prescribed most frequently for twice a day use. However, no clear daily dosage was recorded in 43.9% of those prescribed gabapentin and 27.8% prescribed pregabalin.

**Table 4:** Daily dosing regimen by year for all first prescriptions of gabapentin or pregabalin

**Drug**	**Year**	***n***	**OD**	**BD**	**TDS**	**Other**	**PRN**	**Unknown**
**gabapentin**	**2006**	4,210	279 (6.6)	175 (4.2)	1,812 (43.0)	44 (1.0)	236 (5.6)	1,664 (39.5)
	**2007**	5,433	307 (5.7)	178 (3.3)	2,392 (44.0)	29 (0.5)	217 (4.0)	2,310 (42.5)
	**2008**	6,083	333 (5.5)	184 (3.0)	2,626 (43.2)	46 (0.8)	213 (3.5)	2,681 (44.1)
	**2009**	6,938	374 (5.4)	181 (2.6)	3,073 (44.3)	38 (0.5)	217 (3.1)	3,055 (44.0)
	**2010**	7,207	382 (5.3)	154 (2.1)	3,059 (42.4)	27 (0.4)	179 (2.5)	3,406 (47.3)
**pregabalin**	**2006**	2,719	139 (5.1)	1,336 (49.1)	453 (16.7)	10 (0.4)	79 (2.9)	702 (25.8)
	**2007**	2,979	147 (4.9)	1,509 (50.7)	488 (16.4)	6 (0.2)	68 (2.3)	761 (25.5)
	**2008**	3,652	236 (6.5)	1,690 (46.3)	608 (16.6)	18 (0.5)	94 (2.6)	1,006 (27.5)
	**2009**	4,333	275 (6.3)	2,023 (46.7)	706 (16.3)	15 (0.3)	87 (2.0)	1,227 (28.3)
	**2010**	5,416	328 (6.1)	2,518 (46.5)	822 (15.2)	14 (0.3)	116 (2.1)	1,618 (29.9)

OD once daily, BD twice daily, TDS three times daily, PRN as required.

With most therapies, higher daily doses were used as second- or third-line treatment. The opioids morphine, buprenorphine, dihydrocodeine (not in combination with other analgesics), oxycodone, fentanyl, and meptazinol were usually prescribed following another first-line regimen as were nefopam, duloxetine, lidocaine patches and lower doses of mirtazapine. Lidocaine patches were infrequently used in post-herpetic neuralgia and duloxetine and venlafaxine were prescribed to less than 100 patients (<2%) as a first-line treatment in the painful diabetic neuropathy cohort (Additional file 2).

## Discussion

### Post-herpetic neuralgia and phantom limb pain

This study found an annual incidence per 10,000 person-years adjusted to the 2010 UK age and sex distribution of 3.0 for post-herpetic neuralgia and 0.1 for phantom limb pain, with rates increasing for post-herpetic neuralgia during the study period (Figure 1). This study updates two previous similar incidence estimates based on UK primary care electronic records between 1992 and 2005 [6,7]. Post-herpetic neuralgia decreased between the two earlier study periods from 4.0 (1992–2002) to 2.8 (2002–2005), compared to 3.4 per 10,000 in the current study. When the two most recent studies were age-sex standardised to 2010 levels, a small rise from 2.7 to 3.0 per 10,000 was seen between 2002–2005 and the current study. There were few cases of phantom limb pain with 2010 age-sex adjusted incidences per 10,000 person-years of 0.11 (95%CI 0.07, 0.17) in the current study compared to 0.08 (95%CI 0.04, 0.17) in 2002–2005 suggesting little change.

An annual age-sex adjusted incidence of painful diabetic neuropathy of 3.1 per 10,000 was found. However, the results of the GP questionnaire indicates that this is an over estimate as only 56% of those identified had a final diagnosis of painful diabetic neuropathy. A review of the codes and treatment patterns of those with and without a final diagnosis of painful diabetic neuropathy showed that recording was similar in both groups with none using a specific Read code. Consequently it was not possible to alter the case definition to improve sensitivity. It is feasible that the introduction of the UK Quality and Outcomes Framework (QoF) in 2004 resulted in GPs increasingly making a preliminary diagnosis of painful diabetic neuropathy which is then not confirmed at secondary care. Within this government initiative, primary care physicians are paid to review chronically ill patients, including diabetic patients, with some payment dependent on evidence of checking for complications such as neuropathy. A record of neuropathy testing increased sharply around the time of QoF introduction with one study reporting a rise from 8% of diabetic patient records in the 15 months to April 2003 to 66% in the 15 months to April 2005 with rates of recording then levelling [17]. There has also been increased awareness of neuropathic pain with the introduction and marketing of new licensed treatments and publication of treatment guidelines. This may have resulted in an increase in both the diagnosis of neuropathic pain and the use of treatments such as amitriptyline and gabapentin for less well defined conditions such as neuropathic back pain. The rate of ‘painful diabetic neuropathy’ (as per our definition) increased over the study period from an annual incidence of 2.7 to 3.8 per 100,000 population (Figure 1).

Most other studies assessing the incidence of these neuropathic pain conditions have been within specific sub-populations, such as people with acute herpes zoster, diabetes or post-amputation. One earlier UK general population study reported incidence per 10,000 population of 5.4 for diabetic polyneuropathy and 1.1 for post-herpetic neuralgia [18]. A study of shingles in patients aged 50 years or more (2000–2006) found that 19.5% had post-herpetic neuralgia at one month and 13.7% at three months which would give incidence rates per 10,000 population of 10.2 at one month and 7.2 at three months, which is in-line with this study’s findings given the older population [19].

### Treatment

Amitriptyline was the most commonly prescribed treatment in a first-line therapy for the post-herpetic neuralgia and painful diabetic neuropathy cohorts and the second most frequent after gabapentin in phantom limb pain. Gabapentin was also frequently prescribed as a first-line treatment in the painful diabetic neuropathy cohort and in post-herpetic neuralgia, with the use of both gabapentin and pregabalin increasing over the study period. This demonstrates a maintained shift from predominant use of opioid and non-opioid analgesics in the late 1990’s to use of tricyclic antidepressants and antiepileptics, with initiation of these therapies now evident in primary, rather than secondary, care. This shift appears to be the case across conditions as our painful diabetic neuropathy cohort will have included other neuropathic pain. Compared to the 2002–2005 data, amitriptyline remains a common first-line treatment although use has decreased in post-herpetic neuralgia and phantom limb pain (2002–2005 : 2006–2010, 50% : 39% in post-herpetic neuropathy and 41% : 21% in phantom limb pain). Antiepileptic prescribing has moved from carbamazepine to gabapentin and pregabalin, both of which are now used more frequently in phantom limb pain (2002–2005 versus 2006–2010: 17% versus 38%). Opioid use has increased slightly with tramadol now prescribed more often than co-codamol (codeine phosphate and paracetamol) as a first-line treatment in phantom limb pain. The changes may be due to a combination of an increasing evidence of efficacy in both placebo-controlled and head-to-head trials, education and marketing.

The prescribing analysis period of 2006–2010 was, for the most part, prior to the publication of the current UK and European recommendations on neuropathic pain treatment, although after the publication of key randomised controlled trials for the newer antiepileptics, antidepressants and opioids in neuropathic pain [10,20]. The prescribing of antiepileptics and antidepressants as first-line treatment is consistent with the current recommendations and the shift from carbamazepine to gabapentin, or pregabalin is supported by evidence from clinical trials. The European Federation of Neurological Sciences (EFNS) 2010 guidelines recommends gabapentin, pregabalin, lidocaine plasters and tricyclic antidepressants in post-herpetic neuralgia and duloxetine, gabapentin, pregabalin, tricyclic antidepressants and venlafaxine in painful diabetic neuropathy [10]. The 2010 UK National Institute for Clinical Excellence (NICE) guidelines recommends first-line treatment with duloxetine or, if contraindicated, amitriptyline for painful diabetic neuropathy and amitriptyline or pregabalin as first-line treatment options for other neuropathic pain [20]. Duloxetine, venlafaxine (painful diabetic neuropathy) and topical lidocaine (post-herpetic neuralgia) use was uncommon in this study although there was evidence of efficacy before the study period [10] and all three treatments were included in guidelines as second-line interventions in 2006 [9]. They were not recommended as first-line agents until 2010 [10,20]. The common use of paracetamol first-line in the painful diabetic neuropathy cohort is consistent with guidance in type 1 diabetes from 2004 to 2010 [21] although this recommendation has been superseded. GPs may have followed type 1 guidance until specific guidance for type 2 diabetes was available from NICE in 2008 [22]. Paracetamol is currently recommended for use as a first-line treatment in combination with tramadol in neuropathic pain with acute exacerbations and this may partially explain the common use of both drugs in phantom limb pain [10].

Co-codamol was still one of the five most commonly prescribed first-line treatments in post-herpetic neuralgia and painful diabetic neuropathy and co-dydramol (paracetamol and dihydrocodeine) in post-herpetic neuralgia. While neither of these options is generally recommended as a first line treatment [10] it is not unusual to try conventional analgesics before moving to antidepressants and antiepileptics. The frequent prescription of capsaicin as a first-line treatment for post-herpetic neuropathy (unchanged since 2002–5) is not consistent with current evidence although it is recommended as a second line therapy in the EFNS 2010 guidelines [10].

The analysis of treatments in more than 100 patients showed that the most commonly prescribed daily doses for amitriptyline and pregabalin were those recommended by NICE (10 mg and 150 mg per day respectively) while higher dosage regimens were not uncommon. EFNS recommends a higher daily dose of tricyclic antidepressants (25–150 mg/day) and their suggested dose of gabapentin (1200–3600 mg/day) was prescribed first-line to a minority of the post-herpetic neuralgia and painful diabetic neuropathy cohorts. The analysis of pregabalin dosage regimen did not show a shift from a three to twice a day dosing although this has been suggested as a cost saving exercise. The majority of patients were prescribed one item as a first prescription and one therapeutic category. This is in-line with current guidance although there is some evidence that less than half of patients achieve significant benefit with any single treatment and that combined treatment may be more effective [23]. Patients who received more than one therapeutic category usually received a mixture of an opioid and a non-opioid analgesic rather than combinations with demonstrated efficacy in neuropathic pain, such as gabapentin and opioids or nortriptyline [10]. The proportion of patients who received first-line combination therapy had decreased since the last analysis for post-herpetic neuralgia (44% to 35%), painful diabetic neuropathy cohort (36% to 18%) and phantom limb pain (47% to 27%). Most treatment regimens (monotherapy and combination therapy) were prescribed at first-line (Additional file 2). This may simply be because many patients did not progress to subsequent/later-line therapy.

### Neuropathic back pain and post-operative pain

The inclusion of neuropathic back pain and post-operative pain was considered exploratory, because these are less well defined than the other study conditions with no specific Read code to simplify recording by the GP. The initial strategy for identifying post-operative pain involved identifying records with a code for surgery followed by a record of post-operative pain, plus any marker that this was neuropathic. When few cases were identified, the search focused on breast and hernia surgery, which are associated with post-operative pain [13,24]. The case definition was broadened to include those with a code for surgery followed by a record of neuropathic pain, or a combination of medical and therapy entries indicative of neuropathic pain. Only 49 patients were identified despite the use of a wide case definition. The low patient numbers might partially be due to our method of case selection, for example we would have missed cases if the GP recorded ‘pain’ and used free text to indicate the post-operative or neuropathic nature of that pain. It is also possible that there is under-diagnosis of post-operative pain in primary care or that previous studies have over-ascertained significant long-term post-operative pain. This area therefore warrants additional research.

Neuropathic back pain within the study definition increased over the study period from 49 to 62 per 10,000 patient-years. This cannot be considered to be a true incidence because, without a specific code for neuropathic back pain, the study definition lacked specificity. For example, patients with back pain or radiculopathy treated with tramadol would have been included in the analysis although tramadol is often also used in nociceptive, rather than neuropathic pain. Conversely patients with neuropathic back pain but without a neuropathic code and treated with opioid/non-opioid combinations would not have been identified. Additionally, radicular back pain is not always considered to be neuropathic. The prescribing patterns in the neuropathic back pain cohort differed from the other pain conditions, with tramadol the most frequent first-line therapy and other opioid/non-opioid analgesics commonly prescribed. This may be because of co-existing nociceptive pain for which tramadol efficacy is established [10,25] or indicate that cases of purely nociceptive pain were included in the cohort.

### Strengths and limitations

This study was based solely on primary care records, which provides a picture of routine treatment but has a number of resultant limitations. In particular, not all cases of post-herpetic neuralgia, painful diabetic neuropathy and phantom limb pain were identified using specific disease codes. The incidence of painful diabetic neuropathy is considered a maximum rate as the study definition will include diabetic patients with neuropathic pain which is not related to diabetes or non-painful neuropathy if this was diagnosed at the same time as the patient started a neuropathic pain therapy but for a different indication. The rates of disease we report are those recorded by the GP so any pain not reported to the GP will not be included. Additionally, treatments for pain other than neuropathic pain will have been included in the treatment analysis if they were started at the time of the first neuropathic pain record. More than a third of patients with neuropathic pain are known to have other chronic pain-related disease [26]. We could not identify a first-line therapy for a number of patients possibly because treatment started before a diagnosis was made (phantom limb pain case definition included a record of neuropathic pain at least 3 months after amputation), over the counter medications were used, anti-epileptic treatments used in patients with epilepsy, treatments such as non-steroidal anti-inflammatory drugs were prescribed or when the patients did not require or want a prescription treatment. The validation questionnaire was sent to a small sample and, while the response rate was good, we have no data on the quality of the response and some bias is possible. For example, a record of a working rather than final diagnoses, or inconsistencies between the questionnaire and notes, may affect the likelihood of a reply.

## Conclusion

While treatment for many patients with the neuropathic pain conditions complied with guidelines available at that time, this prescribing was often inconsistent with published evidence of efficacy which is now incorporated into updated guidelines. Use of antiepileptics with demonstrated efficacy as first-line therapy has increased. Unlike post-herpetic neuralgia, painful diabetic neuropathy is difficult to identify from primary care coded records alone, which has implications for future attempts to establish incidence using this method. Few cases of post-operative pain were identified and further research is needed to understand whether this is due to low rates of diagnosis, identification or if published rates include mild intermittent pain.

## Abbreviations

BNF: British national formulary; EFNS: European federation of neurological sciences; (EFNS) NICE: National institute for clinical excellence; PDPN: Painful diabetic neuropathy; PHN: Post-herpetic neuralgia; PLP: Phantom limb pain; QoF: Quality and outcomes framework.

## Competing interests

The study was funded by Pfizer UK, without restriction on publication. Gillian Hall, Henry McQuay, and Steve Morant were funded by Pfizer to design and run the study to answer objectives provided by Pfizer. No payment was made for the development of the manuscript. Gillian Hall and Henry McQuay have received funding for research and consultancy from a number of pharmaceutical companies and charities. Steve Morant has received funding for consultancy from a number of pharmaceutical companies. Dawn Carroll and Zahava Gabriel were both employed by Pfizer at the time of the study and original development of the manuscript.

## Authors’ contributions

GH contributed to all aspects of the study. SM contributed to the analysis and interpretation of data. DC and HM contributed to the design of the study and interpretation of the results. ZG contributed to the conception and design. All authors commented on and approved the manuscript.

## Pre-publication history

The pre-publication history for this paper can be accessed here:

http://www.biomedcentral.com/1471-2296/14/28/prepub

## Supplementary Material

Additional file 1 First-, second-, or third-line treatment for therapies prescribed to more than 100 patients by neuropathic pain condition; n (% with this therapy and dose included in this treatment regimen). Click here for file

Additional file 2 Drugs used in the identification of cases of neuropathic pain (a wider list was used in the treatment analysis). Click here for file
