# Supplementary material for: An observational descriptive study of the epidemiology and treatment of neuropathic pain in a UK general population
Source: BMC Fam Pract. 2013 Feb 26;14:28. doi: 10.1186/1471-2296-14-28 (PMC3599764; doi:10.1186/1471-2296-14-28)
Supplement: Additional file 1 — First-, second-, or third-line treatment for therapies prescribed to more than 100 patients by neuropathic pain condition; n (% with this therapy and dose included in this treatment regimen). [file 1471-2296-14-28-S1.docx]

**Electronic Publication: Drugs used in the identification of cases of neuropathic pain (a wider list was used in the treatment analysis)**

Amitriptyline

Amoxapine

Capsaicin

Clomipramine

Desipramine

Dosulepin

Doxepin

Duloxetine

Imipramine

Ketamine

Lidocaine

Lofepramine

Nortriptyline

Tramadol

Trimipramine

Tryptophan

Venlafaxine

**Only if no history of epilepsy:**

Beclamide

Carbamazepine

Clobazam

Clonazepam

Eslicarbazepine

Ethosuximide

Fosphenytoin

Gabapentin

Lamotrigine

Levetiracetam

Methylphenobarbital

Oxcarbazepine

Phenobarbital

Phenytoin

Pregabalin

Sodium valproate

Sultiame

Tiagabine

Topiramate

Valproate

Vigabatrin
