# Supplementary material for: An observational descriptive study of the epidemiology and treatment of neuropathic pain in a UK general population
Source: BMC Fam Pract. 2013 Feb 26;14:28. doi: 10.1186/1471-2296-14-28 (PMC3599764; doi:10.1186/1471-2296-14-28)
Supplement: Additional file 2 — Drugs used in the identification of cases of neuropathic pain (a wider list was used in the treatment analysis). [file 1471-2296-14-28-S2.docx]

**Electronic only table: First-, second-, or third-line treatment for therapies prescribed to more than 100 patients by neuropathic pain condition; n (% with this therapy and dose included in this treatment regimen)**

| **Drug^a^** | | **Daily dose** | | **Treated patients^b^ *n*** | **1^st^ line**  ***n* (%)** | **2^nd^ line**  ***n* (%)** | **3^rd^ line**  ***n* (%)** | |
| --- | --- | --- | --- | --- | --- | --- | --- | --- |
| 1. **Post-herpetic neuralgia** | | | |  |  |  |  | |
| **Amitriptyline** | **10mg** | | 692 | | 475 (68.6) | 284 (41.0) | | 132 (19.1) |
|  | **15mg** | | 328 | | 223 (68.0) | 138 (42.1) | | 63 (19.2) |
|  | **20mg** | | 224 | | 137 (61.2) | 94 (42.0) | | 59 (26.3) |
|  | **25mg** | | 455 | | 261 (57.4) | 215 (47.3) | | 120 (26.4) |
|  | **50mg** | | 186 | | 90 (48.4) | 74 (39.8) | | 54 (29.0) |
| **Capsaicin** | **0.025% cream** | | 420 | | 177 (42.1) | 154 (36.7) | | 88 (21.0) |
|  | **0.075% cream** | | 393 | | 164 (41.7) | 127 (32.3) | | 90 (22.9) |
| **Carbamazepine** | **200mg** | | 102 | | 42 (41.2) | 48 (47.1) | | 32 (31.4) |
| **Codeine /paracetamol** | **48mg + 3000mg** | | 331 | | 232 (70.1) | 137 (41.4) | | 55 (16.6) |
|  | **64mg + 4000mg** | | 156 | | 103 (66.0) | 65 (41.7) | | 25 (16.0) |
|  | **180mg + 3000mg** | | 558 | | 373 (66.8) | 251 (45.0) | | 150 (26.9) |
|  | **240mg + 4000mg** | | 114 | | 76 (66.7) | 57 (50.0) | | 29 (25.4) |
| **Dihydrocodeine /paracetamol** | **60mg + 3000mg** | | 342 | | 240 (70.2) | 146 (42.7) | | 65 (19.0) |
|  | **80mg + 4000mg** | | 118 | | 73 (61.9) | 51 (43.2) | | 18 (15.3) |
| **Gabapentin** | **300mg** | | 171 | | 78 (45.6) | 67 (39.2) | | 40 (23.4) |
|  | **900mg** | | 263 | | 101 (38.4) | 118 (44.9) | | 82 (31.2) |
| **Lidocaine** | **5% patch** | | 111 | | 23 (20.7) | 22 (19.8) | | 30 (27.0) |
| **Paracetamol** | **3000mg** | | 442 | | 279 (63.1) | 215 (48.6) | | 131 (29.6) |
|  | **4000mg** | | 389 | | 213 (54.8) | 159 (40.9) | | 116 (29.8) |
| **Pregabalin** | **150mg** | | 163 | | 62 (38.0) | 58 (35.6) | | 52 (31.9) |
| **Tramadol** | **300mg** | | 249 | | 120 (48.2) | 111 (44.6) | | 71 (28.5) |

^a^Prescriptions with a daily dose not recorded or not interpretable are not listed. ^b^The number of patients in the total column may be greater than the total across other columns as one treatment could be included in more than one regimen.

| **Table cont.**  **Drug** | **Daily dose** | **Treated patients^b^ *n*** | | | **1^st^ line**  ***n* (%)** | | **2^nd^ line**  ***n* (%)** | | **3^rd^ line**  ***n* (%)** |
| --- | --- | --- | --- | --- | --- | --- | --- | --- | --- |
| **B. Painful diabetic neuropathy** | | |  | |  | |  | |  |
| **Amitriptyline** | **10mg** | | 632 | | 489 (77.4) | | 228 (36.1) | | 119 (18.8) |
|  | **15mg** | | 200 | | 154 (77.0) | | 64 (32.0) | | 38 (19.0) |
|  | **20mg** | | 191 | | 115 (60.2) | | 66 (34.6) | | 55 (28.8) |
|  | **25mg** | | 375 | | 257 (68.5) | | 158 (42.1) | | 88 (23.5) |
|  | **50mg** | | 235 | | 130 (55.3) | | 94 (40.0) | | 88 (37.4) |
| **Buprenorphine** | **120mcg** | | 101 | | 16 (15.8) | | 23 (22.8) | | 17 (16.8) |
|  |  | |  | |  | |  | |  |
| **Capsaicin** | **0.025% cream** | | 199 | | 108 (54.3) | | 56 (28.1) | | 22 (11.1) |
|  | **0.075% cream** | | 139 | | 71 (51.1) | | 43 (30.9) | | 18 (12.9) |
|  |  | |  | |  | |  | |  |
| **Citalopram** | **20mg** | | 155 | | 73 (47.1) | | 62 (40.0) | | 46 (29.7) |
| **Codeine/ paracetamol** | **48mg + 3000mg** | | 293 | | 159 (54.3) | | 128 (43.7) | | 55 (18.8) |
|  | **64mg + 4000mg** | | 111 | | 53 (47.7) | | 42 (37.8) | | 24 (21.6) |
|  | **180mg + 3000mg** | | 503 | | 256 (50.9) | | 205 (40.8) | | 120 (23.9) |
|  |  | |  | |  | |  | |  |
| **Dihydrocodeine/ paracetamol** | **60mg + 3000mg** | | 241 | | 119 (49.4) | | 95 (39.4) | | 56 (23.2) |
|  |  | |  | |  | |  | |  |
| **Fluoxetine** | **20mg** | | 122 | | 64 (52.5) | | 51 (41.8) | | 40 (32.8) |
| **Gabapentin** | **300mg** | | 211 | | 117 (55.5) | | 85 (40.3) | | 52 (24.6) |
|  | **900mg** | | 364 | | 199 (54.7) | | 152 (41.8) | | 127 (34.9) |
|  | **1800mg** | | 145 | | 73 (50.3) | | 70 (48.3) | | 60 (41.4) |
| **Paracetamol** | **3000mg** | | 529 | | 297 (56.1) | | 259 (49.0) | | 156 (29.5) |
|  | **4000mg** | | 437 | | 219 (50.1) | | 177 (40.5) | | 132 (30.2) |
| **Pregabalin** | **150mg** | | 242 | | 120 (49.6) | | 95 (39.3) | | 73 (30.2) |
|  | **300mg** | | 140 | | 54 (38.6) | | 52 (37.1) | | 44 (31.4) |
|  |  | |  | |  | |  | |  |
| **Tramadol** |  | |  | |  | |  | |  |
|  | **300mg** | | 285 | | 119 (41.8) | | 106 (37.2) | | 69 (24.2) |
|  | **400mg** | | 100 | | 31 (31.0) | | 34 (34.0) | | 28 (28.0) |
|  |  | |  |  | |  | |  | |

| **Table cont.**  **Drug** | | **Daily dose** | **Treated patients^b^ *n*** | **1^st^ line**  ***n* (%)** | **2^nd^ line**  ***n* (%)** | **3^rd^ line**  ***n* (%)** |  |
| --- | --- | --- | --- | --- | --- | --- | --- |
| **C. Neuropathic back pain** | | | |  |  |  |  |
| **Amitriptyline** | | **10mg** | | 10737 | 5,759 (53.6) | 3,734 (34.8) | 2,064 (19.2) |
|  | | **15mg** | | 4898 | 2,647 (54.0) | 1,651 (33.7) | 912 (18.6) |
|  | | **20mg** | | 3036 | 1,287 (42.4) | 1,055 (34.7) | 705 (23.2) |
|  | | **25mg** | | 5455 | 2,236 (41.0) | 1,961 (35.9) | 1,424 (26.1) |
|  | | **30mg** | | 971 | 375 (38.6) | 304 (31.3) | 213 (21.9) |
|  | | **37.5mg** | | 1130 | 448 (39.6) | 397 (35.1) | 290 (25.7) |
|  | | **40mg** | | 118 | 26 (22.0) | 29 (24.6) | 31 (26.3) |
|  | | **50mg** | | 2728 | 863 (31.6) | 877 (32.1) | 794 (29.1) |
|  | | **75mg** | | 519 | 171 (32.9) | 161 (31.0) | 131 (25.2) |
|  | | **100mg** | | 409 | 110 (26.9) | 118 (28.9) | 106 (25.9) |
|  | | **150mg** | | 193 | 67 (34.7) | 64 (33.2) | 56 (29.0) |
|  | | **10mg PRN** | | 258 | 139 (53.9) | 94 (36.4) | 52 (20.2) |
| **Aspirin** | | **300mg** | | 186 | 107 (57.5) | 90 (48.4) | 79 (42.5) |
| **Buprenorphine patch** | | **120mcg** | | 2670 | 228 (8.5) | 445 (16.7) | 461 (17.3) |
|  | | **240mcg** | | 2123 | 177 (8.3) | 326 (15.4) | 366 (17.2) |
|  | | **480mcg** | | 1020 | 80 (7.8) | 130 (12.7) | 161 (15.8) |
|  | | **840mcg** | | 492 | 47 (9.6) | 64 (13.0) | 86 (17.5) |
|  | | **1260mcg** | | 212 | 19 (9.0) | 24 (11.3) | 27 (12.7) |
|  | | **1680mcg** | | 121 | 7 (5.8) | 11 (9.1) | 17 (14.0) |
| **Buprenorphine** | | **200mcg** | | 197 | 18 (9.1) | 38 (19.3) | 38 (19.3) |
| **Capsaicin** | | **0.025% cream** | | 2244 | 860 (38.3) | 485 (21.6) | 275 (12.3) |
|  | | **0.075% cream** | | 533 | 146 (27.4) | 103 (19.3) | 70 (13.1) |
| **Carbamazepine** | | **100mg** | | 118 | 40 (33.9) | 41 (34.7) | 22 (18.6) |
|  | | **300mg** | | 138 | 46 (33.3) | 41 (29.7) | 33 (23.9) |
|  | | **200mg** | | 320 | 91 (28.4) | 96 (30.0) | 64 (20.0) |
|  | | **400mg** | | 219 | 87 (39.7) | 80 (36.5) | 71 (32.4) |
| **Citalopram** | | **20mg** | | 3718 | 1,421 (38.2) | 1,518 (40.8) | 1,343 (36.1) |
|  | | **40mg** | | 1573 | 479 (30.5) | 503 (32.0) | 523 (33.2) |
| **Clonazepam** | | **500 mcg** | | 389 | 154 (39.6) | 139 (35.7) | 114 (29.3) |
| **Codeine phosphate** | | **45mg** | | 105 | 32 (30.5) | 43 (41.0) | 32 (30.5) |
|  | | **60mg** | | 659 | 235 (35.7) | 257 (39.0) | 155 (23.5) |
|  | | **90mg** | | 1116 | 450 (40.3) | 440 (39.4) | 221 (19.8) |
|  | | **120mg** | | 1430 | 529 (37.0) | 582 (40.7) | 339 (23.7) |
|  | | **135mg** | | 114 | 33 (28.9) | 54 (47.4) | 31 (27.2) |
|  | | **180mg** | | 1773 | 722 (40.7) | 686 (38.7) | 456 (25.7) |
|  | | **225mg** | | 387 | 44 (11.4) | 70 (18.1) | 56 (14.5) |
|  | | **240mg** | | 320 | 89 (27.8) | 102 (31.9) | 82 (25.6) |
|  | | **30mg PRN** | | 106 | 51 (48.1) | 54 (50.9) | 33 (31.1) |

| **Table cont.**  **Drug** | **Daily dose** | | **Treated patients *n^b^*** | **1^st^ line**  ***n* (%)** | **2^nd^ line**  ***n* (%)** | **3^rd^ line**  ***n* (%)** |
| --- | --- | --- | --- | --- | --- | --- |
| **Codeine/**  **Paracetamol** | | **48mg + 3000mg** | 5539 | 2,725 (49.2) | 2,658 (48.0) | 1,307 (23.6) |
|  |  | **57.6mg + 3600mg** | 240 | 110 (45.8) | 112 (46.7) | 53 (22.1) |
|  | | **60mg + 1000mg** | 142 | 62 (43.7) | 75 (52.8) | 44 (31.0) |
|  | | **64mg + 4000mg** | 2254 | 1,106 (49.1) | 978 (43.4) | 504 (22.4) |
|  | | **90mg + 3000mg** | 527 | 225 (42.7) | 241 (45.7) | 107 (20.3) |
|  | | **120mg + 2000mg** | 209 | 110 (52.6) | 105 (50.2) | 53 (25.4) |
|  | | **120mg + 4000mg** | 1305 | 614 (47.0) | 556 (42.6) | 261 (20.0) |
|  | | **135mg + 2250mg** | 158 | 82 (51.9) | 71 (44.9) | 46 (29.1) |
|  | | **150mg + 2500mg** | 111 | 63 (56.8) | 58 (52.3) | 28 (25.2) |
|  | | **180mg + 3000mg** | 16035 | 8,497 (53.0) | 8,449 (52.7) | 4,950 (30.9) |
|  | | **216mg + 3600mg** | 257 | 111 (43.2) | 110 (42.8) | 71 (27.6) |
|  | | **240mg + 4000mg** | 3579 | 1,669 (46.6) | 1,568 (43.8) | 957 (26.7) |
|  | | **30mg + 500mg PRN** | 275 | 141 (51.3) | 142 (51.6) | 114 (41.5) |
|  | | **8mg + 500mg PRN** | 213 | 115 (54.0) | 132 (62.0) | 68 (31.9) |
| **Dextropropoxyphene /paracetamol** | | **195mg + 1950mg** | 918 | 515 (56.1) | 424 (46.2) | 308 (33.6) |
|  |  | **260mg + 2600mg** | 481 | 253 (52.6) | 198 (41.2) | 143 (29.7) |
|  | | **32.5mg +325mg PRN** | 137 | 95 (69.3) | 80 (58.4) | 64 (46.7) |
| **Dihydrocodeine** | | **60mg** | 136 | 43 (31.6) | 60 (44.1) | 47 (34.6) |
|  | | **90mg** | 147 | 54 (36.7) | 57 (38.8) | 37 (25.2) |
|  | | **120mg** | 823 | 244 (29.6) | 312 (37.9) | 262 (31.8) |
|  | | **135mg** | 1439 | 493 (34.3) | 644 (44.8) | 447 (31.1) |
|  | | **144mg** | 436 | 68 (15.6) | 92 (21.1) | 77 (17.7) |
|  | | **180mg** | 1604 | 435 (27.1) | 594 (37.0) | 494 (30.8) |
|  | | **240mg** | 580 | 136 (23.4) | 190 (32.8) | 151 (26.0) |
|  | | **30mg PRN** | 134 | 51 (38.1) | 74 (55.2) | 40 (29.9) |
| **Dihydrocodeine/ paracetamol** | | **45mg + 2250mg** | 242 | 59 (24.4) | 75 (31.0) | 82 (33.9) |
|  |  | **60mg + 3000mg** | 208 | 98 (47.1) | 93 (44.7) | 48 (23.1) |
|  | | **72mg + 3600mg** | 6883 | 3,385 (49.2) | 3,268 (47.5) | 1,748 (25.4) |
|  | | **80mg + 4000mg** | 150 | 63 (42.0) | 58 (38.7) | 47 (31.3) |
|  | | **144mg + 3600mg** | 2596 | 1,212 (46.7) | 1,109 (42.7) | 645 (24.8) |
|  | | **216mg + 3600mg** | 284 | 85 (29.9) | 92 (32.4) | 76 (26.8) |
|  | | **10mg + 500mg PRN** | 165 | 104 (63.0) | 106 (64.2) | 67 (40.6) |
| **Dosulepin** | | **25mg** | 446 | 207 (46.4) | 152 (34.1) | 104 (23.3) |
|  | | **37.5mg** | 123 | 51 (41.5) | 49 (39.8) | 29 (23.6) |
|  | | **50mg** | 266 | 119 (44.7) | 107 (40.2) | 77 (28.9) |
|  | | **75mg** | 669 | 320 (47.8) | 248 (37.1) | 201 (30.0) |
|  | | **150mg** | 263 | 140 (53.2) | 118 (44.9) | 102 (38.8) |
| **Duloxetine** | | **30mg** | 176 | 25 (14.2) | 30 (17.0) | 38 (21.6) |
|  | | **60mg** | 628 | 126 (20.1) | 130 (20.7) | 128 (20.4) |
| **Escitalopram** | | **5mg** | 132 | 41 (31.1) | 55 (41.7) | 38 (28.8) |
|  |  |  |  |  |  |  |
|  | | **10mg** | 495 | 207 (41.8) | 211 (42.6) | 184 (37.2) |
|  | | **20mg** | 438 | 168 (38.4) | 152 (34.7) | 150 (34.2) |

| **Table cont.**  **Drug** | | **Daily dose** | **Treated patients *n^b^*** | | **1^st^ line**  ***n* (%)** | **2^nd^ line**  ***n* (%)** | | **3^rd^ line**  ***n* (%)** | |
| --- | --- | --- | --- | --- | --- | --- | --- | --- | --- |
| **Fentanyl** | **288mcg** | 1110 | 69 (6.2) | | | 137 (12.3) | | 161 (14.5) | |
|  | **600mcg** | 1449 | 122 (8.4) | | | 230 (15.9) | | 286 (19.7) | |
|  | **1200mcg** | 882 | 82 (9.3) | | | 133 (15.1) | | 161 (18.3) | |
|  | **1800mcg** | 389 | 40 (10.3) | | | 55 (14.1) | | 66 (17.0) | |
|  | **2400mcg** | 206 | 18 (8.7) | | | 29 (14.1) | | 35 (17.0) | |
| **Fluoxetine** | **20mg** | 3326 | 1,515 (45.6) | | | 1,562 (47.0) | | 1,343 (40.4) | |
|  | **40mg** | 1081 | 397 (36.7) | | | 364 (33.7) | | 387 (35.8) | |
|  | **60mg** | 313 | 81 (25.9) | | | 73 (23.3) | | 85 (27.2) | |
| **Gabapentin** | **200mg** | 107 | 20 (18.7) | | | 30 (28.0) | | 26 (24.3) | |
|  | **300mg** | 1729 | 551 (31.9) | | | 487 (28.2) | | 390 (22.6) | |
|  | **600mg** | 408 | 107 (26.2) | | | 118 (28.9) | | 105 (25.7) | |
|  | **900mg** | 2621 | 618 (23.6) | | | 736 (28.1) | | 677 (25.8) | |
|  | **1200mg** | 295 | 65 (22.0) | | | 74 (25.1) | | 80 (27.1) | |
|  | **1800mg** | 888 | 172 (19.4) | | | 209 (23.5) | | 236 (26.6) | |
|  | **2400mg** | 105 | 21 (20.0) | | | 23 (21.9) | | 35 (33.3) | |
|  | **2700mg** | 145 | 20 (13.8) | | | 23 (15.9) | | 27 (18.6) | |
|  | **300mg PRN** | 186 | 53 (28.5) | | | 50 (26.9) | | 36 (19.4) | |
| **Lidocaine** | **5% patch** | 589 | 73 (12.4) | | | 94 (16.0) | | 89 (15.1) | |
| **Lofepramine** | **70mg** | 110 | 47 (42.7) | | | 33 (30.0) | | 23 (20.9) | |
|  | **140mg** | 286 | 103 (36.0) | | | 96 (33.6) | | 80 (28.0) | |
| **Meptazinol** | **800mg** | 117 | 23 (19.7) | | | 36 (30.8) | | 26 (22.2) | |
|  | **1200mg** | 361 | 32 (8.9) | | | 99 (27.4) | | 89 (24.7) | |
| **Mirtazapine** | **15mg** | 688 | 136 (19.8) | | | 151 (21.9) | | 135 (19.6) | |
|  | **30mg** | 946 | 233 (24.6) | | | 270 (28.5) | | 244 (25.8) | |
|  | **45mg** | 494 | 132 (26.7) | | | 105 (21.3) | | 116 (23.5) | |
| **Morphine** | **10mg** | 103 | 8 (7.8) | | | 21 (20.4) | | 27 (26.2) | |
|  | **20mg** | 536 | 55 (10.3) | | | 101 (18.8) | | 132 (24.6) | |
|  | **30mg** | 165 | 24 (14.5) | | | 35 (21.2) | | 43 (26.1) | |
|  | **40mg** | 231 | 24 (10.4) | | | 42 (18.2) | | 51 (22.1) | |
|  | **300mg** | 271 | 18 (6.6) | | | 48 (17.7) | | 45 (16.6) | |
|  | **200mg** | 318 | 23 (7.2) | | | 43 (13.5) | | 61 (19.2) | |
|  | **60mg** | 735 | 82 (11.2) | | | 146 (19.9) | | 175 (23.8) | |
|  | **120mg** | 254 | 44 (17.3) | | | 47 (18.5) | | 52 (20.5) | |
|  | **10mg/5ml PRN** | 156 | 15 (9.6) | | | 44 (28.2) | | 47 (30.1) | |
| **Nefopam** | **90mg** | 181 | 20 (11.0) | | | 42 (23.2) | | 45 (24.9) | |
|  | **135mg** | 188 | 22 (11.7) | | | 54 (28.7) | | 41 (21.8) | |
|  | **180mg** | 289 | 33 (11.4) | | | 66 (22.8) | | 62 (21.5) | |
| **Nortriptyline** | **10mg** | 342 | 120 (35.1) | | | 98 (28.7) | | 69 (20.2) | |
|  | **15mg** | 239 | 132 (55.2) | | | 78 (32.6) | | 42 (17.6) | |
|  | **20mg** | 137 | 43 (31.4) | | | 32 (23.4) | | 32 (23.4) | |
|  | **25mg** | 130 | 46 (35.4) | | | 31 (23.8) | | 22 (16.9) | |
|  | **10mg PRN** | 117 | 49 (41.9) | | | 33 (28.2) | | 20 (17.1) | |

| **Table cont.**  **Drug^a^** | | **Daily dose** | | **Treated patients *n^b^*** | | | **1^st^ line**  ***n* (%)** | **2^nd^ line**  ***n* (%)** | | **3^rd^ line**  ***n* (%)** | |
| --- | --- | --- | --- | --- | --- | --- | --- | --- | --- | --- | --- |
| **Oxycodone** | | **10mg** | | 109 | 7 (6.4) | | | 21 (19.3) | | 17 (15.6) | |
|  | | **20mg** | | 399 | 43 (10.8) | | | 79 (19.8) | | 93 (23.3) | |
|  | | **40mg** | | 418 | 48 (11.5) | | | 76 (18.2) | | 84 (20.1) | |
|  | | **80mg** | | 186 | 14 (7.5) | | | 14 (7.5) | | 31 (16.7) | |
| **Paracetamol** | | **2000mg** | | 142 | 66 (46.5) | | | 64 (45.1) | | 47 (33.1) | |
|  | | **3000mg** | | 9029 | 3,921 (43.4) | | | 4,125 (45.7) | | 3,202 (35.5) | |
|  | | **3600mg** | | 572 | 224 (39.2) | | | 237 (41.4) | | 189 (33.0) | |
|  | | **4000mg** | | 8685 | 3,331 (38.4) | | | 3,409 (39.3) | | 2,704 (31.1) | |
|  | | **4800mg** | | 158 | 71 (44.9) | | | 65 (41.1) | | 48 (30.4) | |
|  | | **500mg PRN** | | 308 | 162 (52.6) | | | 165 (53.6) | | 140 (45.5) | |
| **Paracetamol/tramadol** | | **1462.5mg + 168.75mg** | | 104 | 18 (17.3) | | | 29 (27.9) | | 17 (16.3) | |
|  |  | **1950mg + 225mg** | | 398 | 108 (27.1) | | | 109 (27.4) | | 74 (18.6) | |
|  | | **2600mg + 300mg** | | 227 | 48 (21.1) | | | 69 (30.4) | | 65 (28.6) | |
| **Paroxetine** | | **20mg** | | 531 | 329 (62.0) | | | 287 (54.0) | | 237 (44.6) | |
|  | | **30mg** | | 225 | 129 (57.3) | | | 100 (44.4) | | 93 (41.3) | |
|  | | **40mg** | | 143 | 77 (53.8) | | | 56 (39.2) | | 56 (39.2) | |
| **Pregabalin** | | **50mg** | | 322 | 81 (25.2) | | | 68 (21.1) | | 54 (16.8) | |
|  | | **75mg** | | 212 | 61 (28.8) | | | 65 (30.7) | | 42 (19.8) | |
|  | | **100mg** | | 373 | 105 (28.2) | | | 98 (26.3) | | 87 (23.3) | |
|  | | **150mg** | | 1757 | 454 (25.8) | | | 478 (27.2) | | 383 (21.8) | |
|  | | **200mg** | | 199 | 38 (19.1) | | | 41 (20.6) | | 46 (23.1) | |
|  | | **225mg** | | 138 | 24 (17.4) | | | 33 (23.9) | | 27 (19.6) | |
|  | | **300mg** | | 891 | 140 (15.7) | | | 194 (21.8) | | 205 (23.0) | |
|  | | **400mg** | | 112 | 15 (13.4) | | | 17 (15.2) | | 20 (17.9) | |
|  | | **450mg** | | 152 | 20 (13.2) | | | 24 (15.8) | | 35 (23.0) | |
|  | | **600mg** | | 500 | 61 (12.2) | | | 76 (15.2) | | 99 (19.8) | |
| **Sertraline** | | **50mg** | | 903 | 298 (33.0) | | | 344 (38.1) | | 284 (31.5) | |
|  | | **100mg** | | 754 | 269 (35.7) | | | 233 (30.9) | | 214 (28.4) | |
|  | | **200mg** | | 127 | 41 (32.3) | | | 32 (25.2) | | 30 (23.6) | |
| **Tramadol** | | **50mg** | | 188 | 93 (49.5) | | | 64 (34.0) | | 45 (23.9) | |
|  | | **75mg** | | 314 | 155 (49.4) | | | 112 (35.7) | | 58 (18.5) | |
|  | | **100mg** | | 1631 | 846 (51.9) | | | 590 (36.2) | | 314 (19.3) | |
|  | | **150mg** | | 2427 | 1,301 (53.6) | | | 820 (33.8) | | 391 (16.1) | |
|  | | **300mg** | | 19271 | 9,724 (50.5) | | | 6,720 (34.9) | | 3,309 (17.2) | |
|  | | **200mg** | | 6072 | 2,868 (47.2) | | | 2,216 (36.5) | | 1,194 (19.7) | |
|  | | **225mg** | | 3072 | 1,687 (54.9) | | | 1,034 (33.7) | | 459 (14.9) | |
|  | | **350mg** | | 106 | 44 (41.5) | | | 34 (32.1) | | 24 (22.6) | |
|  | | **360mg** | | 401 | 224 (55.9) | | | 135 (33.7) | | 62 (15.5) | |
|  | | **400mg** | | 3632 | 1,341 (36.9) | | | 1,175 (32.4) | | 800 (22.0) | |
|  | | **450mg** | | 261 | 127 (48.7) | | | 75 (28.7) | | 36 (13.8) | |
|  | | **50mg PRN** | | 7012 | 3,929 (56.0) | | | 2,692 (38.4) | | 1,307 (18.6) | |
| **Venlafaxine** | | **75mg** | | 637 | 276 (43.3) | | | 251 (39.4) | | 197 (30.9) | |
|  | | **150mg** | | 745 | 345 (46.3) | | | 284 (38.1) | | 264 (35.4) | |
|  | | **225mg** | | 101 | 25 (24.8) | | | 24 (23.8) | | 24 (23.8) | |

^a^Prescriptions with a daily dose not recorded or not interpretable are not listed. ^b^The number of patients in the total column may be greater than the total across other columns as one treatment could be included in more than one regimen.
